# Enhancement of superconductivity and phase diagram of Ta-doped Kagome superconductor CsV_3_Sb_5_

**DOI:** 10.1038/s41598-024-59518-1

**Published:** 2024-04-26

**Authors:** Jinjin Liu, Qing Li, Yongkai Li, Xinwei Fan, Jun Li, Peng Zhu, Hanbin Deng, Jia-Xin Yin, Huaixin Yang, Jianqi Li, Hai-Hu Wen, Zhiwei Wang

**Affiliations:** 1 Centre for Quantum Physics, Key Laboratory of Advanced Optoelectronic Quantum Architecture and Measurement (MOE), School of Physics, Beijing Institute of Technology, Beijing, 100081 People’s Republic of China; 2 National Laboratory of Solid State Microstructures and Department of Physics, Collaborative Innovation Center of Advanced Microstructures, Nanjing University, Nanjing, 210093 People’s Republic of China; 3 Beijing National Laboratory for Condensed Matter Physics, Institute of Physics, Chinese Academy of Sciences, Beijing, 100190 People’s Republic of China; 4 Beijing Key Lab of Nanophotonics and Ultrafine Optoelectronic Systems, Beijing Institute of Technology, Beijing, 100081 People’s Republic of China; 5 Material Science Center, Yangtze Delta Region Academy of Beijing Institute of Technology, Jiaxing, 314011 People’s Republic of China; 6 Department of Physics, Southern University of Science and Technology, Shenzhen, Guangdong People’s Republic of China

**Keywords:** Materials science, Physics

## Abstract

Kagome superconductors AV_3_Sb_5_ (A = K, Rb, and Cs) have attracted enormous interest due to the coexistence of charge density wave (CDW) order, unconventional superconductivity (SC) and anomalous Hall effect (AHE). In this paper, we reported an intensive investigation on Cs(V_1−*x*_Ta_*x*_)_3_Sb_5_ single crystals with systematic Ta doping. Ta was confirmed to be doped into V-site in the Kagome layer from both single crystal X-ray diffraction structural refinement and scanning transmission electron microscopy observation. The highest Ta doping level was found to be about 16%, which is more than twice as much as 7% in Nb-doped CsV_3_Sb_5_. With the increase of Ta doping, CDW order was gradually suppressed and finally vanished when the doping level reached to more than 8%. Meanwhile, superconductivity was enhanced with a maximum critical temperature (*T*c) of 5.3 K, which is the highest *T*c in the bulk crystal of this Kagome system at ambient pressure so far. The *μ*_0_*H*_c2_(T) behavior demonstrates that the system is still a two-band superconductor after Ta doping. Based on the electrical transport measurement, a phase diagram was set up to exhibit the evolution of CDW and SC in the Cs(V_1−*x*_Ta_*x*_)_3_Sb_5_ system. These findings pave a new way to search for new superconductors with higher *T*c in the AV_3_Sb_5_ family and establish a new platform for tuning and controlling the multiple orders and superconducting states.

## Introduction

The newly discovered Kagome superconductor *A*V_3_Sb_5_ (*A* = K, Rb, Cs) family attracted tremendous attention because it is considered to be a good platform for studying electron correlation, lattice geometry and nontrivial band topology. Different kinds of techniques have already revealed that this material exhibits many exotic properties such as charge density wave (CDW), anomalous Hall effect (AHE), and unconventional superconductivity (SC)^1–10^. Generally speaking, *A*V_3_Sb_5_ undergoes a CDW transition at *T*_CDW_ about 78, 103, and 94 K for *A* = K, Rb, and Cs, respectively, accompanied with a three-dimensional (3D) chiral 2 × 2 × 2 charge order^11–16^, which attributes to the Kagome structural distortion of two possible types, namely Star of David (SoD) and Tri-hexagonal (TrH)^17,18^. At low temperature, superconductivity with a critical temperature (*T*c) from about 0.9 to 3 K has been reported^2,4,19^. Nuclear magnetic resonance (NMR) and muon spin rotation/relaxation technique (μSR) have evidenced the unconventional superconductor for CsV_3_Sb_5_ (CVS) on account of multiple s-wave and nodeless superconducting gaps^20,21^. Although thermal conductivity measurements have exhibited node superconducting gaps^22^, ultrahigh-resolution and low-temperature angle-resolved photoemission spectroscopy (ARPES) observation clearly revealed a nodeless and nearly isotropic superconducting gap in CVS very recently^23^.

The CDW and SC can be tuned by applying external pressure. Wang et al. reported that CDW was suppressed gradually with the increase of pressure and finally vanished, while superconductivity showed nonmonotonic behavior and two SC domes appeared with the maximum *T*c enhanced to about 8 K^24^. Similar phenomena were reported independently by several other groups as well^25–29^. Besides pressurization, chemical doping with hole or electron is also an effective way to tune CDW and SC. For example, CDW was suppressed very rapidly in Sn-doped CsV_3_Sb_5−*x*_Sn_*x*_ and almost vanished when the doping level *x* > 0.05. At the same time, superconductivity exhibits a nonmonotonic evolution with the Sn doping resulting in two “domes” peaked at 3.6 and 4.1 K^30,31^. Yang et al. and Liu et al. reported independently that Ti doping can kill the CDW very quickly, however, the evolution of SC exhibited quite different behavior^32,33^. *T*c behaved V-shape in Ref.^32^ and single-dome-shape in Ref.^33^ as the Ti concentration increased. In addition, Cr and Mo were also reported to be successfully doped into V-site, and both caused the suppression of SC, but CDW showed completely opposite evolution: Cr suppressed CDW while Mo promoted it^34,35^. Our recent work on Nb-doped CVS demonstrated a different situation, with *x* increased to the solution limit of 7%, *T*c was monotonously enhanced up to a maximum of 4.45 K, while CDW was not completely suppressed^36^. Further ARPES study and band calculation revealed an unconventional competition mechanism between SC and CDW in light of the band structure modification near the Fermi surface^37^.

In this letter, we reported the successful growth of Ta-doped Cs(V_1−*x*_Ta_*x*_)_3_Sb_5_ single crystals with the highest doping level up to *x* = 0.16. We investigated evolution of CDW and superconductivity with various doping levels, although samples with the low doping ratio of *x* ≤ 0.04 have been reported^35^. The Ta doping level was also more than twice as much as in our previous Nb-doped CVS. Both TEM and XRD analysis demonstrated that Ta was effectively substituted for V-site in the Kagome layer. CDW transitions were suppressed gradually with the increase of doping level, and finally vanished when the doping level *x* is higher than 0.10. Meanwhile, superconductivity was enhanced gradually with the increase of Ta content and the highest *T*c of 5.3 K was observed in the *x* = 0.16 sample, which is the highest value at ambient pressure in the bulk samples of the *A*V_3_Sb_5_ family so far. A comprehensive phase diagram has been established to illustrate the evolution of CDW and SC in the Cs(V_1−*x*_Ta_*x*_)_3_Sb_5_ system.

### Experiments

#### Crystal growth

Cs(V_1−*x*_Ta_*x*_)_3_Sb_5_ (*x* = 0, 0.04, 0.05, 0.08, 0.1, 0.14 and 0.16) single crystals were synthesized from Cs bulk (Alfa Aesar, 99.8%), V piece (Aladdin, 99.97%), Ta powder (Alfa Aesar, 99.99%) and Sb shot (Alfa Aesar, 99.9999%) via a flux method as described in our previous report. Flux mixtures containing 5 mol percent Cs(V_1−*x*_Ta_*x*_)_3_Sb_5_ were loaded into a crucible, and then placed in a quartz tube in an Ar-filled glove box. After the quartz tube was sealed, it was heated slowly to 1000 °C and maintained for 24 h, followed by cooling down to 200 °C at the rate of 3 °C/h. Finally, the furnace was brought down to room temperature with the power switched off. To remove the flux, the obtained samples need to be soaked in deionized water. At last, high-quality single crystals with hexagonal shapes were obtained. We would like to point out that the highest doping level of Ta was *x* = 0.16, even if we tried to raise the nominal concentration to *x* = 0.5.

### Measurement method

A specimen of Cs(V_0.86_Ta_0.14_)_3_Sb_5_ was used for the single crystal X-ray crystallographic analysis, which was taken from a Bruker D8 QUEST single crystal diffractometer at 293 K, equipped with the APEX III software and Mo radiation. The structure was solved and refined by using the Bruker SHELXTL Software Package to obtain the information on crystal structure. After that, Cs(V_1−*x*_Ta_*x*_)_3_Sb_5_ single crystals were structurally and chemically characterized by X-ray diffraction (XRD) using a Bruker D8 Advance diffractometer with Cu-Kα radiation, and energy dispersive X-ray spectroscopy (EDX) equipped on a JEOL scanning electron microscope (SEM, JSM-7500F) to confirm the content of Ta, respectively.

A physical property measurement system (PPMS, Quantum Design) was used for electronic transport measurements with the temperature from 300 K down to 1.8 K. Cs(V_1−*x*_Ta_*x*_)_3_Sb_5_ samples were measured using a five-terminal configuration, in which the longitudinal resistivity (*ρ*_xx_) and the Hall resistivity (*ρ*_yx_) can be measured simultaneously. Temperature dependence of DC magnetic susceptibility was measured in a SQUID magnetometer (Quantum Design MPMS).

Before transmission electron microscope (TEM) analysis, the single crystalline foils with an average thickness of around 50 nm were mechanically exfoliated from the bulk crystal using adhesive tape, and then transferred to the copper grid with the aid of Crystalbond adhesives and acetone. An aberration-corrected JEOL JEM-ARM200CF transmission electron microscope (TEM) was employed to acquire high angle annular dark field (HAADF) image, selected area electron diffraction pattern, and energy dispersive X-ray spectroscopy (EDX) mapping, operated at 200 kV.

## Discussion

### Structure characterization

**Figure 1:**
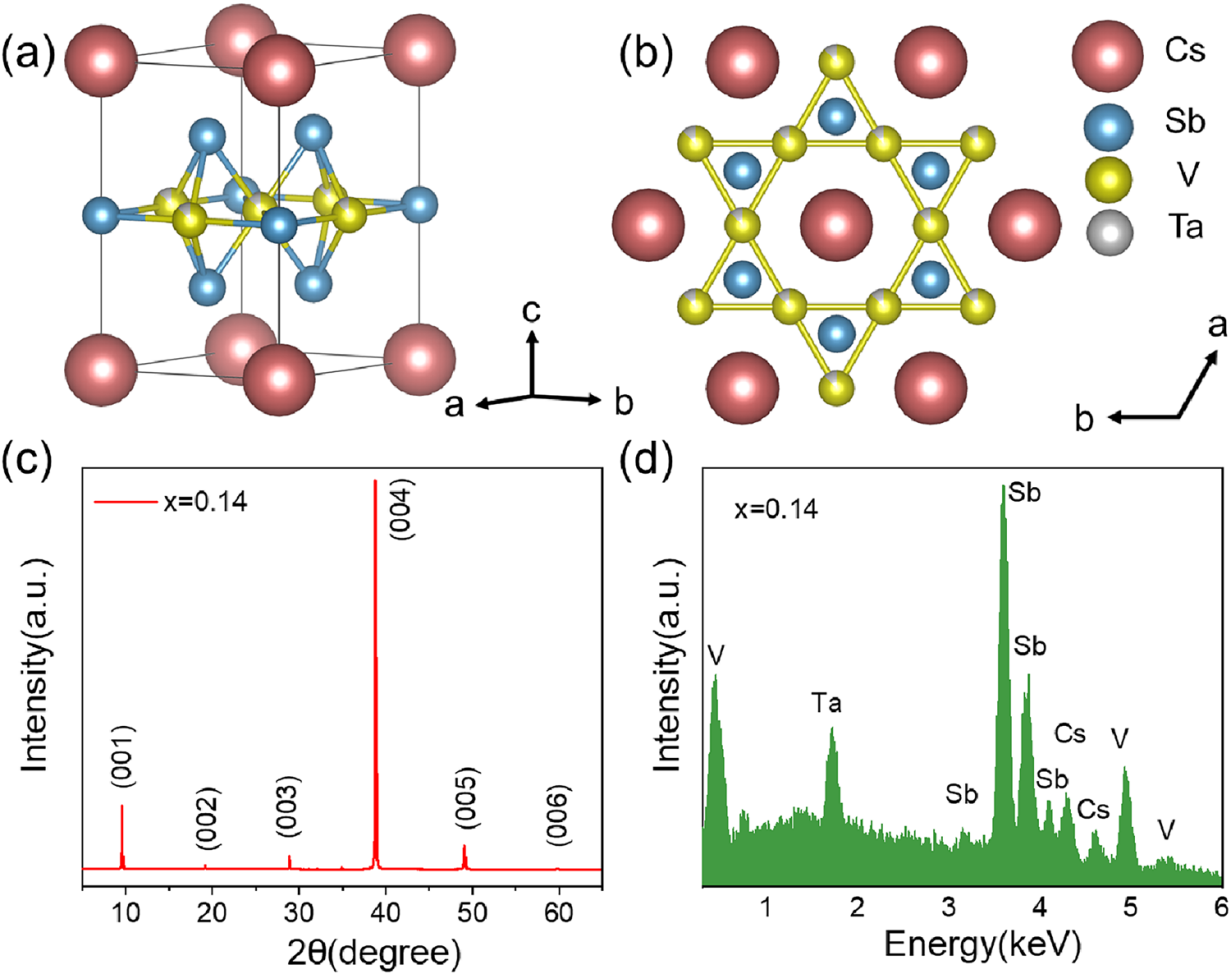
Structure and chemical composition of Cs(V_1−*x*_Ta_*x*_)_3_Sb_5_ crystals. (**a**) Side view and (**b**) top view of Cs(V_1−*x*_Ta_*x*_)_3_Sb_5_ crystal structure. (**c**) XRD pattern of Cs(V_0.86_Ta_0.14_)_3_Sb_5_ single crystal with (00*l*) reflections. (**d**) EDS results of Cs(V_0.86_Ta_0.14_)_3_Sb_5_ sample, the presence of Ta peak confirms Ta was doped into CVS.

A series of Ta-doped Cs(V_1−*x*_Ta_*x*_)_3_Sb_5_ (CVTS) single crystals with *x* = 0, 0.04, 0.05, 0.08, 0.10, 0.14, and 0.16 were synthesized for the first time. Utilizing the SHELXTL software package for single crystal X-ray diffraction analysis (see section “Method”), the crystal structure of CVTS was obtained, as illustrated in Fig. 1a,b. The CVTS samples maintain the same hexagonal structure as the parent phase, with Ta atoms successfully occupying the V sites as expected. X-ray diffraction pattern on *x* = 0.14 sample, as shown in Fig. 1c, also confirmed that the crystal structure can be well indexed with hexagonal structure with the space group of P6/mmm, which is the same as CVS single crystal. Comprehensive structural details are summarized in Tables 1, 2. Notably, the lattice parameters *a*(= *b*) and *c*, were calculated to be 5.5587 Å and 9.3020 Å, respectively, show a discernible expansion compared to the parent CsV_3_Sb_5_ (*a* = 5.4949 Å and *c* = 9.3085 Å). This indicates Ta doping primarily enlarges the *ab* plane while having a relatively small impact on the *c*-axis direction. EDS results in Fig. 1d show a clear Ta peak, which means the successful doping of Ta. In addition, subsequent measurements of electronic transport and magnetic properties showed that the superconducting transition temperature and CDW transition temperature changed gradually, also indicating that Ta had been successfully doped. We would like to point out that the doping concentration *x* adopted in the whole text reflect the actual Ta content determined through EDS analysis, and the doping limit of Ta in CVTS is about 16%, i.e. *x* = 0.16.

**Table 1:** Crystal data and structural refinement for Cs(V_0.86_Ta_0.14_)_3_Sb_5_.

Empirical formula	Cs(V_0.86_Ta_0.14_)_3_Sb_5_
Formula weight (g/mol)	949.44
Temperature	293(2) K
Crystal system	hexagonal
Space group	P 6/m m m
Unit cell dimensions	
a (Å)	5.5587 (12)
b (Å)	5.5587 (12)
c (Å)	9.302 (3)
α	90°
β	90°
γ	120°
Volume (Å^3^)	248.92 (13)
Z	1
Density (g/cm^3^)	6.334
Absorption coefficient (mm^−1^)	23.801
F(000)	400
R_1_, wR_2_	0.0577; 0.1660

**Table 2:** Atomic coordinates and equivalent isotropic atomic displacement parameters (Å^2^) for Cs(V_0.86_Ta_0.14_)_3_Sb_5_.

Atom	x/a	y/b	z/c	U (eq)
Sb1	0.666667	0.333333	0.25561 (11)	0.0218 (8)
Sb2	0.0	0.0	0.5	0.0208 (8)
V1	0.5	0.5	0.5	0.0179 (9)
Ta1	0.5	0.5	0.5	0.0179 (9)
Cs1	0.0	0.0	0.0	0.0364 (9)

**Figure 2:**
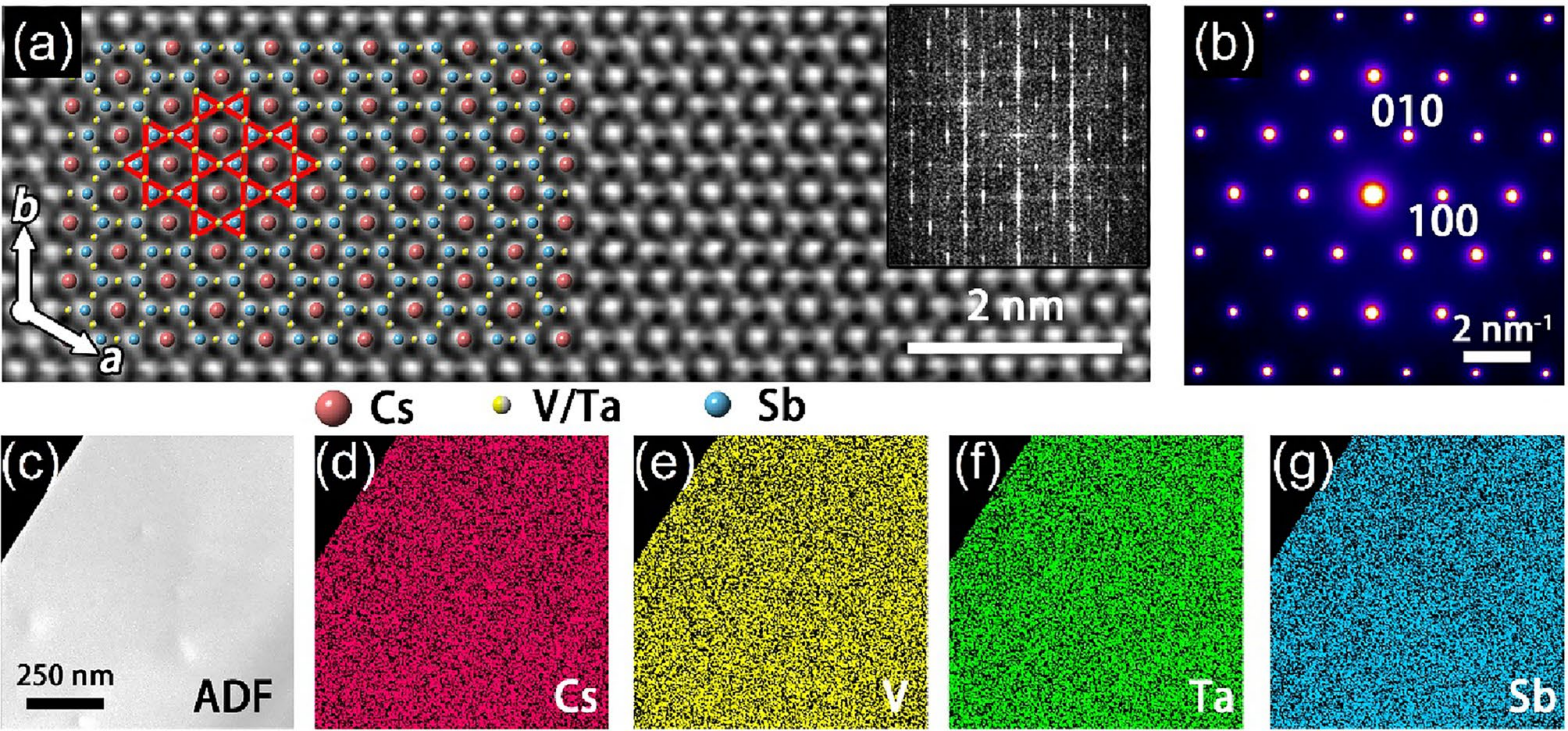
Microstructure characterization of Cs(V_1−*x*_Ta_*x*_)_3_Sb_5_. (**a**) Atomic resolution HRTEM images of CVTS, the inset is FFT pattern. (**b**) electron diffraction pattern taken along the [001] axis direction. (**c**) ADF of CVTS single crystal. (**d**–**g**) elemental mapping of Cs, V, Ta, and Sb elements, respectively.

Atomic-resolution STEM and HAADF images of Cs(V_0.86_Ta_0.14_)_3_Sb_5_ which were taken along the [001] axis direction at room temperature, as shown in Fig. 2a, provide further details on the lattice structure. Given the specimen thickness is relatively thin, the brightness of the atomic columns in the HAADF images scales with the specimen thickness and the constituent elements of atomic number Z (approximately ~ Z^1.7^). Therefore, the relatively heavier elements like Cs and Sb appear brighter than V. Despite Ta being the heaviest among the constituent elements, its low concentration results in negligible contrast alteration. The derived structural model superimposed on the STEM image confirms the isostructure with CsV_3_Sb_5_, and the Kagome lattice can be clearly identified. Both the fast Fourier transform (FFT) (insert in Fig. 2a) and electron diffraction pattern (Fig. 2b) exhibit hexagonal symmetry devoid of any superstructural feature. Besides, to further confirm the successful doping of Ta into the CsV_3_Sb_5_ lattice, the EDX elemental mapping was carried and shown in Fig. 2c–g, validates the presence of Ta dopants and homogeneous distribution.

### Superconductivity, CDW order and their competition

**Figure 3:**
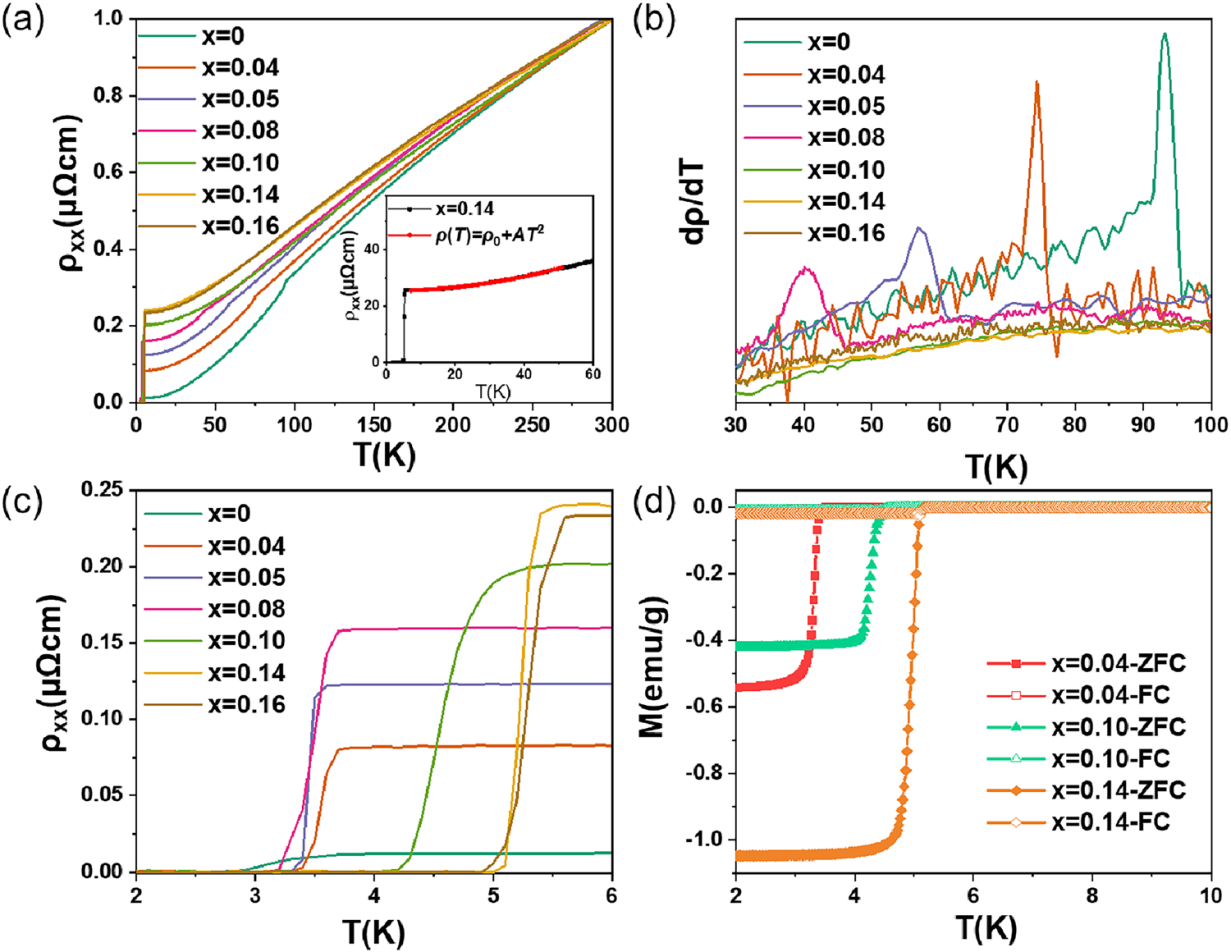
Electrical and magnetic properties of Cs(V_1−*x*_Ta_x_)_3_Sb_5_. (**a**) Temperature dependence of longitudinal resistivity from 2 to 300 K. The inset shows the quadratic temperature dependence from 7 to 50 K. (**b**) Temperature dependence of d*ρ*/d*T* from 30 to 100 K to illustrate CDW transitions. (**c**) *ρ*(*T*) curves around SC transition temperature. (**d**) Temperature dependence of magnetic susceptibility for CVTS measured with the applied field of 5 Oe, both ZFC and FC curves are presented.

Electronic transport and magnetic properties measurements were performed to elucidate the interaction between SC and CDW order. Figure 3 shows temperature dependence of resistivity and magnetization of CVTS, one can see a clear competition between SC and CDW with the increasing of Ta content. As the Ta increases, CDW transition temperature progressively decreases from 93.6 K for parent CVS to 40.5 K for sample with *x* = 0.08, and then faded away completely in samples with the doping content of *x* = 0.10, 0.14 and 0.16, as shown in Fig. 3a. This behavior is more visible from the d*ρ*/d*T* curves, as shown in Fig. 3b, which suggests that Ta doping can weaken CDW effectively and ultimately suppress it. Meanwhile, the resistivity at normal state increased with the increase of doping level, this is because the scattering effect could become stronger after Ta doping. Concurrently, superconducting transition temperature *T*c was significantly increased, as indicated in Fig. 3c, here *T*c is defined as the temperature corresponding to the midpoint of the resistivity drop. With the increasing of Ta, *T*c increased gradually and reached to 5.3 K for *x* = 0.16 sample, which is the highest *T*c observed in the bulk of AV_3_Sb_5_ family at ambient pressure so far. Since the highest *T*c was observed in the sample with the highest Ta content, and neither peak nor saturation was observed as Ta doping, we can expect that *T*c could be higher if one can introduce more Ta into CVS by any other methods. To further check superconductivity of CVTS, temperature dependence of magnetization measurement was conducted under an applied magnetic field of 5 Oe from 2 to 10 K. Both the zero-field-cooled (ZFC) and field-cooled (FC) results are shown in Fig. 3d. One can see distinct diamagnetic transitions for all measured samples, which affirmed the occurrence of SC in CVTS. We would like to point out that the diamagnetic data here cannot reflect the superconducting volume fraction accurately since we focused on the evolution of Tc and the applied magnetic field is perpendicular to the ab-plane. The higher Ta samples have higher *T*c, consistent with that in resistivity measurement. The sharp transitions in both *ρ*(T) and *M*(T) curves indicate the high quality of our CVTS crystals. In addition, in the low temperature region(7–50 K) for *x* = 0.14, the ρ(*T*) curve can be well fitted using the formula ρ(*T*) = ρ_0_ + *AT*^2^ as shown in the inset of Fig. 3a, where ρ_0_ is the residual resistivity, *AT*^2^ term originates from the electron–electron. The fitting gives ρ_0_ = 25.4 μΩ cm, *A* = 3.1 × 10^−3^ μΩ cm K^−2^. The electron–electron scattering process dominates low-temperature resistivity. The quadratic relationship indicates the normal Fermi liquid behavior^37^.

**Figure 4:**
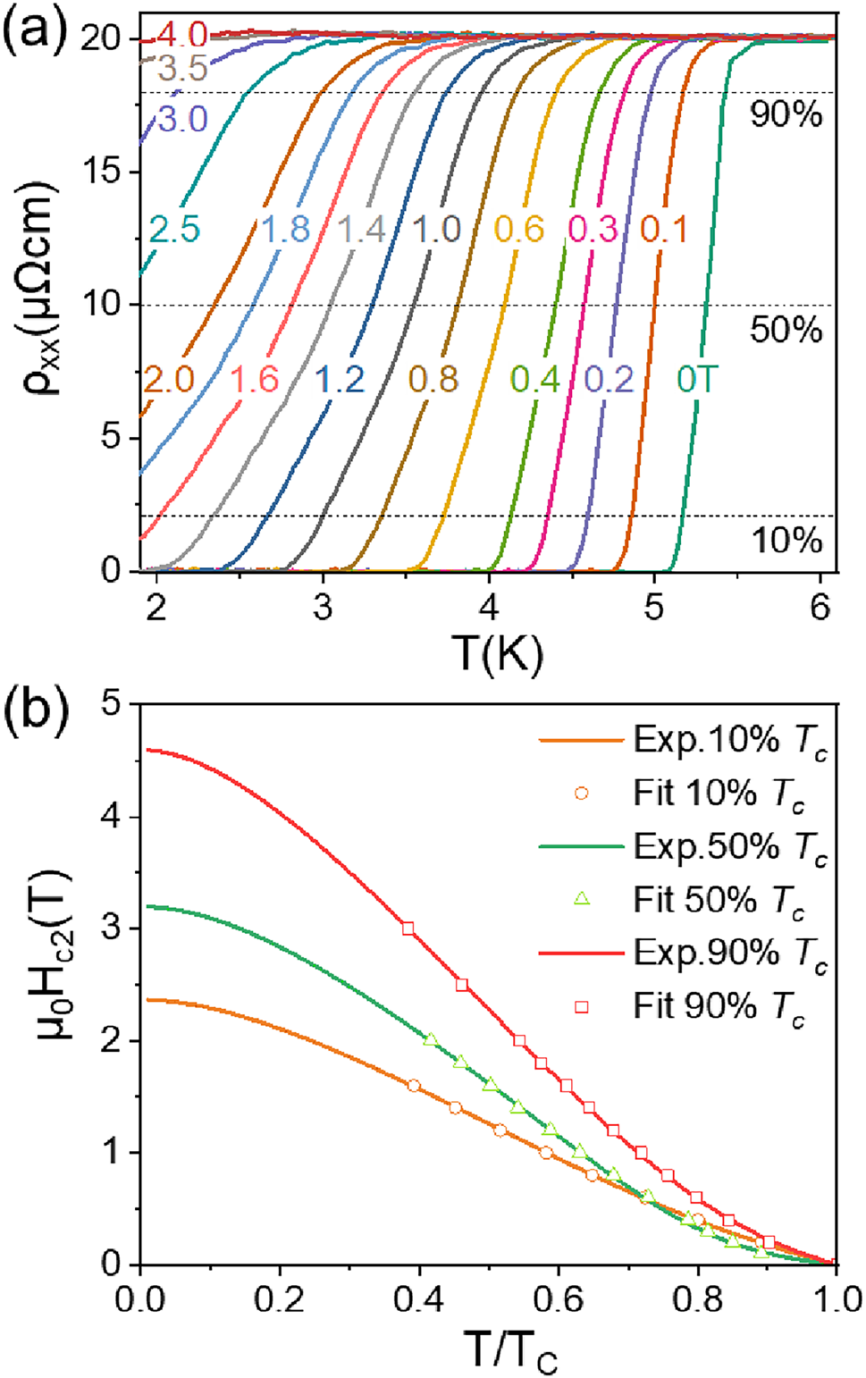
Temperature dependence of longitudinal resistivity under various magnetic fields. (**a**) *ρ*(*T*) curves of Cs(V_0.84_Ta_0.16_)_3_Sb_5_ measured under the applied magnetic field up to 4 T, the current was applied in the *ab* plane and the magnetic field applied along the *c* axis. (**b**) The temperature dependence of the upper critical field extracted from the resistivity curves. The data are fitted well by two band theory.

To examine the evolution of SC under applied magnetic field, we measured the temperature dependence of resistivity at varying magnetic fields on *x* = 0.14 sample with the fields perpendicular to the *ab* plane. Figure 4a presents the *ρ*(T) curves measured from 2 to 6 K under various magnetic fields up to 4 T. One can clearly see a gradual suppression of *T*c with the magnetic field increasing. There is still a SC transition sign around 2 K even the magnetic field was applied as high as 3.5 T. But when the field was applied to 4 T, almost no SC transition was detected. To better analyze the resistive transitions and determine upper critical field *μ*_0_*H*_c2_, the 90%, 50% and 10% levels of normal-state resistivity *ρ*_N_ (shown by dashed lines) are taken to mark the transition. The difference between these three criteria gives an idea about the uncertainty in determining *μ*_0_*H*_c2_. The summary of *μ*_0_*H*_c2_ is plotted in Fig. 4b. The two-band theory^38^ fits all the three sets of data well and yields *μ*_0_*H*_c2_(0) to be 4.6 T, 3.2 T, and 2.4 T, corresponding to 90%, 50%, and 10% criteria, respectively. The detailed fitting process was described in Supplementary file. According to the equation of coherence length $\xi =\sqrt{{\Phi }_{0}/2\pi {\mu }_{0}{H}_{c2}}$, we can estimate the $\xi$ to be 11.7 nm, 10.1 nm, and 8.5 nm, respectively, where ${\Phi }_{0}$ is the magnetic flux quantum.

**Figure 5:**
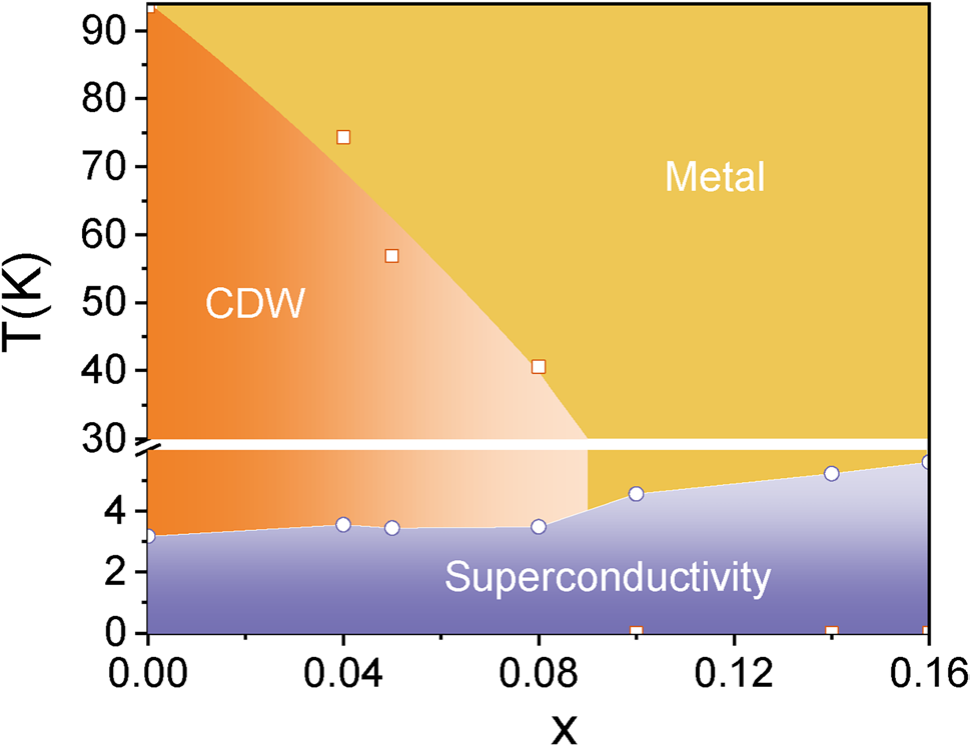
Phase diagram of the Cs(V_1−*x*_Ta_*x*_)_3_Sb_5_ crystals. *T*_CDW_ decreased gradually and finally vanished when *x* is more than 0.1, while *T*_c_ increased significantly with *x* increasing, shows an obvious competition between CDW and SC.

Figure 5 summarize the phase diagram of CVTS single crystals established from the resistivity measurements and the effect of Ta doping on CDW and SC is presented. It is evident that *T*_CDW_ decreased monotonically with increasing Ta content, and was completely suppressed when *x* is more than 0.1. While superconductivity was enhanced with the *T*c gradually increased spontaneously. Interestingly, the enhancement of SC has two distinct regions: when *x* is less than 0.08, i.e. CDW order is coexisted with SC, *T*c enhanced slowly; however, *T*c increased more quickly after CDW was completely suppressed, i.e. in samples with *x* more than 0.1. *T*c does not get saturated as the Ta content increasing. Since the doping limit is 16% in our study by using flux method to grow CVTS crystals, if one can raise the doping limit by using any other different crystal growth techniques, we can expect to obtain higher *T*c sample. This competition behavior between CDW and SC seems similar to that in Nb-doped, Ti-doped and Sn-doped CVS. What’s different is that CDW was not completely suppressed in Nb-doped case^36^, while there appeared two distinct SC regions in Ti-doped^32,33^ and Sn-doped cases^30,31^. These differences reflect complicated competition mechanism between CDW and SC. Nb and Ta are electrically neutral doping and will not introduce additional charge in principle, but since the doping limit of Nb (7%) is smaller than that of Ta (16%), Nb is not enough to suppress CDW completely in Nb-doped CVS. However, Ti or Sn doping will introduce additional electron or hole, which can tune the Fermi level more effectively than Nb and Ta doping. This complicated competition of CDW and SC was also seen in our previous work on ARPES, STM and magnetization measurements^23,32,39–42^.

## Conclusions

High-quality single crystals of Ta-doped Cs(V_1−*x*_Ta_*x*_)_3_Sb_5_ were synthesized with the highest doping level of *x* = 0.16, and the competition between CDW and SC was investigated from electronic transport measurements. With the increasing of Ta, the superconducting critical temperature enhanced gradually and reached to a maximum of 5.3 K, which is the highest *T*c in the bulk of this system at ambient pressure so far. Meanwhile, CDW order became weaker and weaker, and finally suppressed completely when *x* is more than 0.1. The upper critical field *μ*_0_*H*_c2_(0) was increased after Ta doping and estimated to be about 4.6 T, its temperature dependent behavior can be well characterized by two-band theory. Our work provides a platform to study the competition mechanism between SC and CDW in Kagome superconductors, as well as provides a new idea for exploring exotic Kagome superconducting materials with higher *T*c.

### Supplementary Information

Supplementary Information.
