# Supplementary material for: Enhancement of superconductivity and phase diagram of Ta-doped Kagome superconductor CsV3Sb5
Source: Sci Rep. 2024 Apr 26;14:9580. doi: 10.1038/s41598-024-59518-1 (PMC11052999; doi:10.1038/s41598-024-59518-1)
Supplement: Supplementary file 1 — Supplementary Information. [file 41598_2024_59518_MOESM1_ESM.docx]

**Support information for “Enhancement of superconductivity and phase diagram of Ta-doped Kagome superconductor CsV_3_Sb_5_”**

Jinjin Liu^1,4^, Qing Li^2^, Yongkai Li^1,4,5^, Xinwei Fan^2^, Jun Li^3,*^, Peng Zhu^1,4,5^, Hanbin Deng^6^, Jiaxin Yin^6^, Huaixin Yang^3^, Jianqi Li^3^, Hai-Hu Wen^2,*^, Zhiwei Wang^1,4,5,*^

^1^Centre for Quantum Physics, Key Laboratory of Advanced Optoelectronic Quantum Architecture and Measurement (MOE), School of Physics, Beijing Institute of Technology, Beijing 100081, P. R. China.

^2^National Laboratory of Solid State Microstructures and Department of Physics, Collaborative Innovation Center of Advanced Microstructures, Nanjing University, Nanjing 210093, P. R. China

^3^Beijing National Laboratory for Condensed Matter Physics, Institute of Physics, Chinese Academy of Sciences, Beijing 100190, P. R. China

^4^Beijing Key Lab of Nanophotonics and Ultrafine Optoelectronic Systems, Beijing Institute of Technology, Beijing 100081, P. R. China

^5^Material Science Center, Yangtze Delta Region Academy of Beijing Institute of Technology, Jiaxing, 314011, P. R. China

^6^ Department of Physics, Southern University of Science and Technology, Shenzhen, Guangdong, P. R. China

*Email: [zhiweiwang@bit.edu.cn](mailto:zhiweiwang@bit.edu.cn); [junli@iphy.ac.cn](mailto:junli@iphy.ac.cn); hhwen@nju.edu.cn

**Part 1: Two-band process:**

Now we process the calculation of the upper critical field *H_c_*_2_ applied along the *c* axis of a hexagonal crystal. The simpler case of Usadel equations is used to fit:

$2\omega f_{1}-D_{1}^{\alpha\beta}\Pi_{\alpha}\Pi_{\beta}f_{1}=2\Delta_{1},$ (1)

$2\omega f_{2}-D_{2}^{\alpha\beta}\Pi_{\alpha}\Pi_{\beta}f_{2}=2\Delta_{2},$ (2)

$\Delta_{m}=2\pi T\sum_{\omega>0}^{\omega_{D}} \sum_{m} \lambda_{mm^{'}}f_{m^{'}}(\mathbf{r},\omega)$ (3)

where $D_{m}^{\alpha\beta}$ is electronic diffusivity tensors, $\Pi$=▽+2πiA$/\phi_{0}$, **A** is the vector potential, $\phi_{0}$ is the flux quantum, $\Delta_{m}$ are order parameters. The function *f* depends on the coordinates **r** and Matsubara frequency $\omega$. There is the matrix of the BCS superconducting coupling constants $\lambda_{mm^{'}}=\lambda_{mm^{'}}^{(ep)}-\mu_{mm^{'}}$, where $\lambda_{mm^{'}}^{(ep)}$ are electron-phonon constants, and $\mu_{mm^{'}}$ is the matrix of the Coulomb pseudopotential. Due to in-plane isotropic diffusivities, $D_{m}^{\alpha\beta}=D_{m}\delta_{\alpha\beta}$, so we obtain

$\begin{aligned} f_{m}\left( x,\omega\right)=\frac{\Delta_{m}\left( x \right)}{\left( \omega+\frac{\pi HD_{m}}{\phi_{0}} \right)}, \\ \Delta_{m}(x)=\tilde{\Delta}_{m}\exp\left( -\pi Hx^{2}/\phi_{0} \right) \end{aligned}$ (4)

Inserting Eqs. (4) into the gap equation (3) yields two linear equations for ${\overset{\sim}{\Delta}}_{1}$ and ${\overset{\sim}{\Delta}}_{2}$

$2\pi T\sum_{\omega>0}^{\omega_{D}} \frac{1}{\omega+\Omega}=\ln\frac{2\gamma\omega_{D}}{\pi T}-U(\frac{\Omega}{2\pi T})$ (5)

$U\left( x \right)=\psi\left( \frac{1}{2}+x \right)-\psi(\frac{1}{2})$ (6)

where $\psi$(*x*) is the di-gamma function.

$\begin{matrix} & {\overset{\sim}{\Delta}}_{1}=\lambda_{11}[l-U(h)]{\overset{\sim}{\Delta}}_{1}+\lambda_{12}[l-U(\eta h)]{\overset{\sim}{\Delta}}_{2}, \\ & {\overset{\sim}{\Delta}}_{2}=\lambda_{22}[l-U(\eta h)]{\overset{\sim}{\Delta}}_{2}+\lambda_{21}[l-U(h)]{\overset{\sim}{\Delta}}_{1} \end{matrix}$ (7)

where *l* = ln(2g$\omega_{D}$/p*T*), *h* = *H_c_*_2_*D*_1_/2$\phi_{0}$*T*, and $\eta$ = *D*_2_/*D*_1_, the diagonal terms λ_11_ and λ_22_ quantify the intraband superconducting coupling, and off-diagonal terms λ_12_ and λ_21_ describe the interband coupling. The solvability condition of Eqs. (7) gives an equation for *H_c_*_2_, in which it is convenient to express $\omega_{D}$ via *Tc* using equation 1 - $\lambda_{+}$*l_c_ +* *w*$l_{c}^{2}$ *=*0, and $T_{c0}=1.14\omega_{D}exp[-\frac{\lambda_{+}-\lambda_{-}}{2w}]$, where $\lambda_{\pm}=\lambda_{11}\pm\lambda_{22}$, and ${w=\lambda_{11}\lambda}_{22}-{\lambda_{12}\lambda}_{21}$, $\lambda_{0}={(\lambda_{-}^{2}+4\lambda_{12}\lambda_{21})}^{1/2}$.

Therefore, the equation for *H_c_*_2_ takes the form

*y*=*a*_0_[(log *t* + *U*(*h*))][(log *t* +*U*(*ηh*)) + *a*_2_[(log *t* + *U*(*ηh*))] + *a*_1_[(log *t* + *U*(*h*))] (8)

where *a*_0_=2*w*/λ_0_, ${a_{1}=1+\lambda_{-}/\lambda}_{0}$, ${a_{2}=1-\lambda_{-}/\lambda}_{0}$,

Next, we obtain the value of 90%, 50%, 10%*T*c from the results of temperature vs. resistivity curve under different magnetic field, i.e. experimental data (*t*, *h*).

when *y*=0 from Eqs. (8), we can obtain the theoretical data of (*T, H*).

We should adjust the parameters λ_11_, λ_22_, λ_12_, λ_21_, and *η* to make fit well by using MATLAB software.

Finally, for 90%*T*c, λ_11_ = 0.82, λ_22_=0.6, λ_12_= λ_21_=0.35, and *η*=6.5; for 50%*T*c, λ_11_ = 1.25, λ_22_=0.65, λ_12_= λ_21_=0.40, and *η*=34.0; for 10%*T*c, λ_11_ = 1.25, λ_22_=0.68, λ_12_= λ_21_=0.40, and *η*=20.0.

The two-band theory fits all the three sets of data well and yields *μ*_0_H*_c_*_2_(0) to be 4.6 T, 3.2 T, and 2.4 T, corresponding to 90%, 50%, and 10% criteria, respectively. The more detailed fitting process was reported [Phys. Rev. B, 67, 184515 (2003)].
